# Database citation in supplementary data linked to Europe PubMed Central full text biomedical articles

**DOI:** 10.1186/2041-1480-6-1

**Published:** 2015-01-05

**Authors:** Şenay Kafkas, Jee-Hyub Kim, Xingjun Pi, Johanna R McEntyre

**Affiliations:** European Molecular Biology Laboratory, European Bioinformatics Institute (EMBL-EBI), Wellcome Trust Genome Campus, Hinxton, Cambridge CB10 1SD UK

**Keywords:** Text mining, Supplementary data, Accession number, Molecular biology databases

## Abstract

**Background:**

In this study, we present an analysis of data citation practices in full text research articles and their corresponding supplementary data files, made available in the Open Access set of articles from Europe PubMed Central. Our aim is to investigate whether supplementary data files should be considered as a source of information for integrating the literature with biomolecular databases.

**Results:**

Using text-mining methods to identify and extract a variety of core biological database accession numbers, we found that the supplemental data files contain many more database citations than the body of the article, and that those citations often take the form of a relatively small number of articles citing large collections of accession numbers in text-based files. Moreover, citation of value-added databases derived from submission databases (such as Pfam, UniProt or Ensembl) is common, demonstrating the reuse of these resources as datasets in themselves. All the database accession numbers extracted from the supplementary data are publicly accessible from http://dx.doi.org/10.5281/zenodo.11771.

**Conclusions:**

Our study suggests that supplementary data should be considered when linking articles with data, in curation pipelines, and in information retrieval tasks in order to make full use of the entire research article. These observations highlight the need to improve the management of supplemental data in general, in order to make this information more discoverable and useful.

## Background

Biomolecular and literature databases are a vital resource for the scientific community. Linking these resources enables scientists to access, analyse and process the data comprehensively. One way to link these resources is to identify accession numbers as specific database citations in text. Accession number annotation in full text has been tackled in a variety of ways at various points in the publication lifecycle. While some publishers tag (structurally annotate) accession numbers in the text of articles as a part of their production process, this is not something done comprehensively across all publishers [1]. In the absence of machine-actionable citation data, text mining [1–4] can be used to annotate accession numbers automatically across large volumes of published research articles. One such study is our recent work on the citation of three major submission databases (ENA, UniProt, PDBe) within the Open Access subset of Europe PMC (http://europepmc.org/). In this study, we investigated to what extent, (1) publishers provide structurally annotated accession numbers in full text, (2) text mining extends publisher annotations and (3) text mining contributes to literature–database cross links. Our results show that text mining can significantly enrich publishers’ annotations and contribute to literature–database cross links (see [1] for details).

Although the annotation of many types of accession numbers is now part of the routine processing of full text articles in Europe PMC, the extent of data citation in supplementary data has yet to be explored. Supplementary data is unstructured and therefore the content is basically undiscoverable via the usual retrieval methods that operate only on the article narrative. However, finding database citations in supplementary data could be useful for the deep integration of literature and databases and potentially helpful for curators [5]. Moreover, as reported in a recent study, which focuses on mining genetic variations from literature, supplementary materials were identified as a critical source of genetic mutations [6]. Here, we extend our previous study on the analysis of database citation in narrative of the full text articles to supplementary data in order to understand whether supplementary data is useful for linking articles to the biomolecular databases. To the best of our knowledge, this is the first study on the analysis of data citation in supplementary data. In this study: We extended the Whatizit-Accession Number Annotation (Whatizit-ANA) module to annotate database citations to a total of ten biological databases and revised the extraction rules and patterns. We analysed and compared the distribution of the database citations in the body of the Open Access article set (OA-ePMC articles) and their associated supplementary data files for the ten databases. All the database accession numbers extracted from the supplementary data are publicly accessible from http://dx.doi.org/10.5281/zenodo.11771.

## Materials and methods

### Literature and biomedical databases used

#### Literature

**Figure 1:**
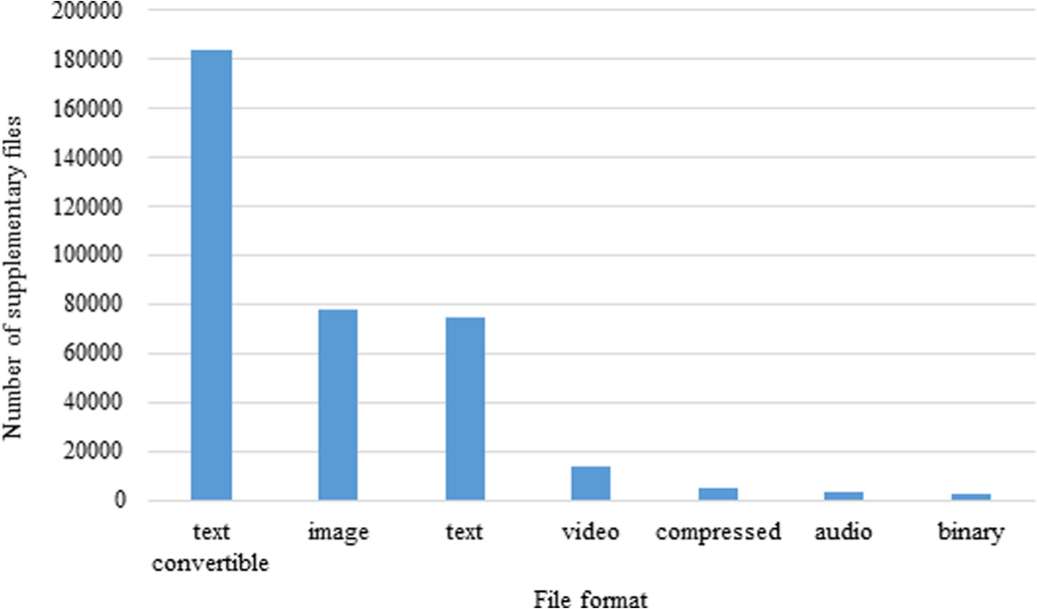
**Distribution of supplementary data by file formats.** This figure describes distribution of supplementary files linked to the Europe PMC open access full text articles by different file formats. The “text convertible” format covers the formats which can be convertible to text such as pdf, xml, html and xsl.

The full text articles and their supplementary files used in this study were gathered from the OA-ePMC set. This open access article set is available on the Europe PMC FTP site and the linked supplementary data files are available via the Europe PMC RESTful web service (http://europepmc.org/restfulwebservice). We decided to reanalyse the set of OA-ePMC articles that we used in our previous study in order for the results to be directly comparable (http://europepmc.org/ftp/oa/AccNoAnalysisData/AnnotatedData/). This set contains 410,364 full text articles in XML format [1]. It is formed by filtering out the articles which were published before 1990 since in this historical set, accession number citations are rare. We identified 361,937 supplementary files that belong to these articles in various formats. The distribution of these files according to the file formats is shown in Figure 1. This shows that the majority of the files have formats such as Portable Document Format (PDF) and Microsoft Word (DOC) and Microsoft Excel (XLS) that can be converted into text.

A three-step pre-processing was applied to the gathered supplementary files: (1) screening out the supplementary files that are not easily convertible to text such as image, audio and movie file types (filtering is done based on MIME types/subtypes that can be extracted from the file link in the full text XML, within the </supplementary-material > element). (2) screening out text supplementary files that are unlikely to contain accession numbers (e.g. source code files) (filtering is done based using known file extensions for source code) and (3) employing Apache Tika [7] to extract the text content from the remaining files. The final set of supplementary data included a total of 213,245 supplementary files either in text or text convertible format linked to 68,995 of the 410,364 OA-ePMC articles.

#### Biological databases

We used ten major biological databases in this study. Three of these databases are primary databases and the other seven databases are added-value (secondary) databases.

#### Primary databases

Primary databases accept direct submissions of de novo data. The following primary databases were used in this study:

- The European Nucleotide Archive (ENA, http://www.ebi.ac.uk/ena/)
- ArrayExpress (http://www.ebi.ac.uk/arrayexpress/)
- Protein Data Bank, Europe (PDBe, http://www.ebi.ac.uk/pdbe/)

#### Added-value databases

Added-value or secondary databases collect or present data as curated sets or summaries based on primary data submissions. The following added-value databases were used in this study:

- The Protein families database (Pfam, http://pfam.sanger.ac.uk/)
- Universal Protein knowledgebase (UniProt, http://www.uniprot.org/)
- Reference Sequence (RefSeq, http://www.ncbi.nlm.nih.gov/RefSeq/)
- Reference Single Nucleotide Polymorphism (RefSNP, http://www.ncbi.nlm.nih.gov/SNP/)
- Ensembl (http://www.ensembl.org/index.html)
- Online Mendelian Inheritance in Man (OMIM, http://www.omim.org/)
- InterPro (http://www.ebi.ac.uk/interpro/)

### Annotating database citations

Database citations in publications were annotated by the text-mining method used in our previous study [1]. This method mainly uses Whatizit-Accession Number Annotation (Whatizit-ANA) module [1, 8] where a set of extraction rules and patterns were applied with contextual cues for recognising database citations. These patterns are shown in Table 1.

**Table 1:** **Extraction patterns and contextual cues for databases**

Database	Patterns	Contextual cues
ENA	[A-Z][0–9]{5}; [A-Z]{2}[0–9]{6}; [A-Z]{3}[0–9]{5}; [A-Z]{4}[0–9]{8,10}; [A-Z]{5}[0–9]{7}	genbank, gen, ddbj, embl
UniProt	[A-N,R-Z][0–9][A-Z][A-Z, 0–9][A-Z, 0–9][0–9]; [O,P,Q][0–9][A-Z, 0–9][A-Z, 0–9][A-Z, 0–9][0–9]	swissprot, sprot, uniprot
PDBe	[0–9][A-Z, 0–9]{3}	pdb
InterPro	IPR[0–9]{6}	interpro
Pfam	PF(AM)?[0–9]{5}	hmm, family, pfam
ArrayExpress	E-[A-Z]{4}-[0–9]+	arrayexpress
OMIM	[0–9]{6}	omim
Ensembl	ENS[A-Z]*G[0–9]{11}+	ensembl
RefSeq	(AC|AP|NC|NG|NM|NP|NR|NT|NW|NZ|XM|XP|XR|YP|ZP|NS)_([A-Z]{4})*[0–9]{6,9}(?:[.][0–9]+)?	refseq
RefSNP	RS[0–9]{5,9}	snp

For this study, we extended our annotation method by:

1. Adding extraction rules and patterns to include ten database types (compared to the first version, accession number annotation is extended to four additional databases: Ensembl, RefSeq, RefSNP and OMIM).
2. Revising the validation step by replacing the previous accession number validator with a new one based on the global EBI Search web service (http://www.ebi.ac.uk/Tools/webservices/services/eb-eye). This new validator covers more databases (e.g. InterPro and Ensembl) and is more robust.

This new Whatizit-ANA module has been integrated into the core Europe PMC infrastructure and is available via Whatizit web site and web service (http://www.ebi.ac.uk/webservices/whatizit).

## Results and discussion

### Performance assessment of the Whatizit-ANA Module

We used the same sets of gold standards (for ENA, UniProt and PDBe) used in our previous study for assessing the performance differences between the previous and current versions of the Whatizit-ANA module. The results presented in Table 2 show that the current version of the module is better than the previous version (F-score values of > 96% for UniProt and PDB and >77% for ENA) (please refer to [1] for our performance evaluation). This is due to the improvement in the validation component that we used in the new version of our tool. This new validation component is capable of validating accession numbers more accurately, resulting in lower numbers of missed accession numbers [see Table 2, the new system misses fewer accession numbers (false negatives) and hence identifies higher number of accession numbers (true positives) compared to the old one]. For example, in the article with PMCID1892096, the UniProt citation P09372 was missed (false negative) using the old version of the tool, however it was annotated correctly with the new version.

**Table 2:** **Performance assessment results of the Whatizit ANA module**

Database	Evaluation	#TP		#FP		#FN		Precision (%)		Recall (%)		F-score (%)	
		New	Old	New	Old	New	Old	New	Old	New	Old	New	Old
**ENA**	**Automatic**	276	267	10	7	170	181	96.50	97.45	61.88	59.60	75.41	73.96
	**Manual**	286	274	0	0	170	181	100	100	62.72	60.22	77.10	75.17
**UniProt**	**Automatic**	574	569	28	8	39	39	95.35	98.61	93.64	93.59	94.49	96.03
	**Manual**	601	577	1	0	39	39	99.83	100	93.91	93.67	96.78	96.73
**PDBe**	**Automatic**	568	529	32	30	12	50	94.67	94.63	97.93	91.36	96.27	92.97
	**Manual**	620	559	0	0	12	50	100	100	98.10	91.79	99.04	95.72

FP: False Positive, FN: False Negative, Old: Old Whatizit-ANA settings, New: New Whatizit-ANA settings.

Manual and automatic evaluation: In the automatic evaluation; we estimated the performance of the tool by assuming that publisher-supplied accession numbers in the articles are a gold standard for annotation. However, when we manually analysed the false positive annotations provided from our pipeline, we realised that the accession numbers provided in articles (the annotations that we assumed as gold standard in the automatic evaluation) might not be always complete or correct. Therefore, the annotations made by our tool, which were not already annotated in the article, were deemed false positives by the automatic evaluation, however, such annotations could be reassigned as true positives on manual inspection.

### Distribution of database citations

**Figure 2:**
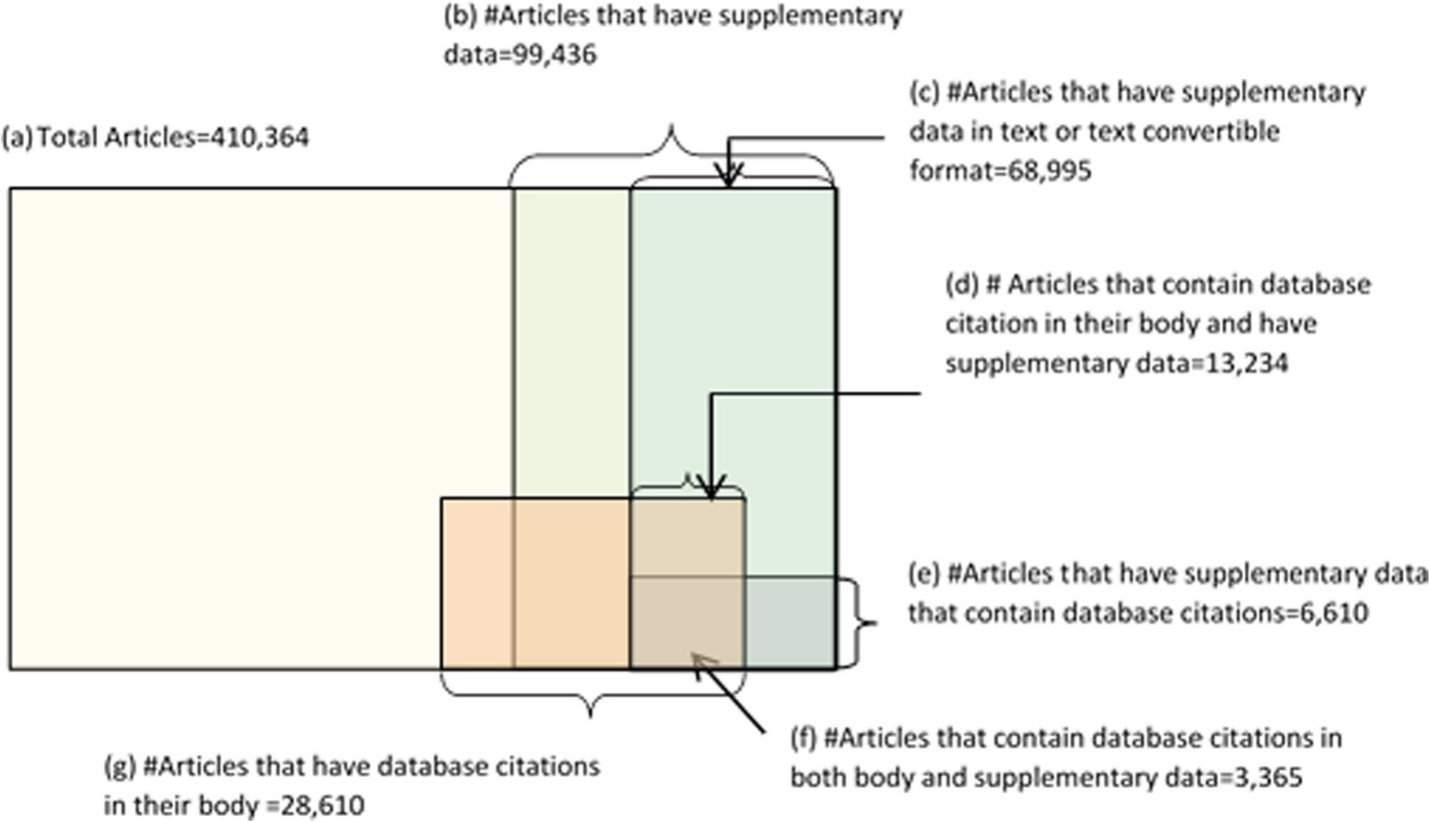
**Distribution of database citations in the OA-ePMC articles.** This figure describes distribution of database citations in the Europe PMC open access full text articles.

Figure 2 shows the distribution of database citation in the 410,364 OA-ePMC articles and their supplementary data. The analysis reveals that 16.8% of articles (68,995/410,364; Figure 2 (c)) have supplementary data in either text or text convertible format. Only, 3,365 of these 68,995 articles (3,365/410,364; 0.82%; Figure 2 (f)) contain database citations in both their body and supplementary data.

### Analysis of database citation in article body and supplementary data

In the full set of 410,364 OA-ePMC articles, 28, 610 (6.97%, Figure 2 (g)) of the article bodies contain database citations. Of the 213,245 supplementary files that we can mine, 10,179 (4.77%) contain database citations. Table 3 shows the distribution of the database citations in the bodies of these articles and supplementary files.

**Table 3:** **Distribution of database citations in article body and supplementary data by databases in the OA-ePMC set**

Database	Supplementary data	Article body	Ratio	Shared citations
Ensembl	1,292,198	1,152	1,121.70	23 (0.002%)
RefSeq	2,540,260	2,864	886.96	178 (0.007%)
InterPro	564,956	639	884.13	77 (0.014%)
UniProt	2,972,519	9,387	316.66	540 (0.018%)
Pfam	924,624	2,968	311.53	435 (0.047%)
RefSNP	2,443,679	31,061	78.67	3,849 (0.16%)
ENA	3,390,319	125,534	27.01	4,167 (0.12%)
PDBe	197,850	44,269	4.47	2,805 (1.42%)
ArrayExpress	2,377	1,565	1.52	53 (2.23%)
OMIM	2,400	2,779	0.86	19 (0.80%)

Clearly, there are many more references to databases in the supplementary data compared to article bodies except for OMIM. This is likely due to the role of the article body in highlighting key data points, and the supplementary files containing complete datasets (e.g. Results from high-throughput analyses that have to be summarized in the main article are provided in supplementary files).

Interestingly, citations of added-value databases are much higher than for primary databases in supplementary files, in contrast to the situation in article narratives, where added-value databases are cited less frequently than primary databases. This suggests that these added-value database records are themselves reused as datasets in their own right. That is to say, a list of accession numbers in a supplemental data file represents a derived dataset used by the authors of the article in the course of their work.

Only a tiny portion of accession numbers is shared in the article body and supplementary material. This indicates that supplementary material should be considered in addition to article body for linking articles to databases, in curation process or in text analytics.

All the database citations extracted from the supplementary files are available from http://dx.doi.org/10.5281/zenodo.11771.

### Distribution of database citations in article bodies and their supplementary data over time

We thought it might be useful to compare database citation trends in article bodies and their supplementary data over time. Therefore, we focused on the articles that have supplementary data either in text or text convertible format only. 68,995 of 410,364 OA-ePMC articles (Figure 2 (c)) are identified as such articles along with their 213,245 supplementary files in text/text convertible format.

**Figure 3:**
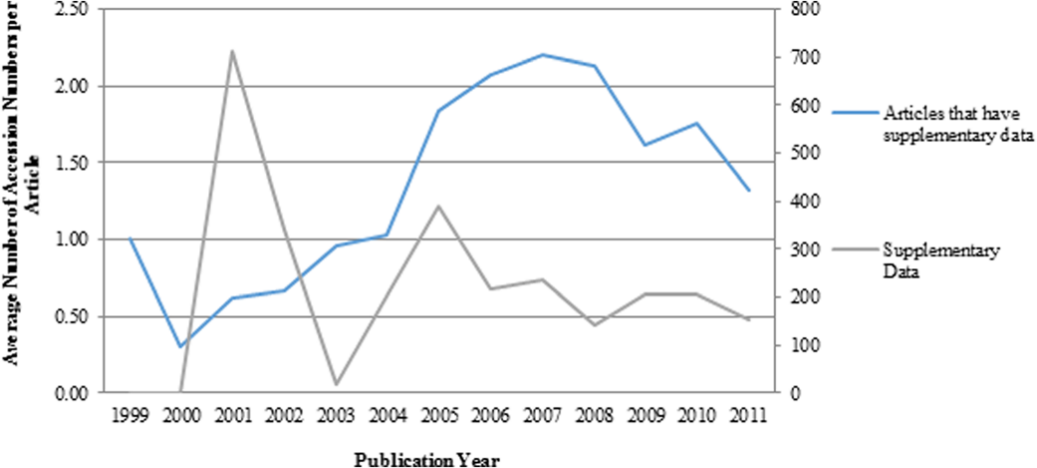
**Distribution of average number of database citations over years.** Articles with supplementary data (left axis), Supplementary data (right axis). This figure describes distribution of average number of database citations over years in supplementary data and in the bodies of articles which have supplementary data.

Figure 3 shows the distribution of the average number of accession numbers identified in the bodies and supplementary files of these articles. On average, the number of citations in the supplementary data is significantly higher than the number of citations in the article bodies. This is perhaps not surprising since authors tend to cite only the key data in the article bodies and provide a larger set of data in supplementary files.

**Figure 4:**
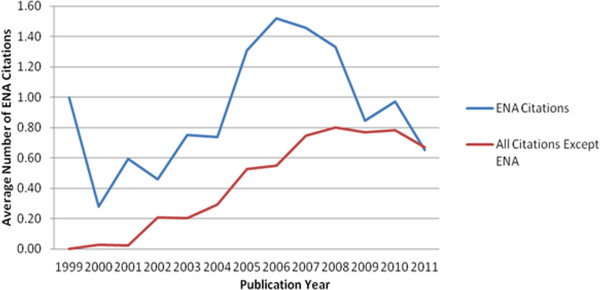
**Average number of database citations in article bodies by including and excluding ENA.** This figure describes distribution of average number of database citations in article bodies by excluding and including ENA citations.

As can be seen from Figure 3, there is a peak in 2007 in the average number of citations in article bodies. A further analysis done on the average number of citations shows that ENA citations are the main source of this trend (see Figure 4).

We further investigated the profiles of database citations in the supplementary data compared to the article bodies. Table 4 shows that 5% of articles are responsible for the majority of data citations found in supplementary data files. This indicates a propensity to have large collections of accession numbers in supplementary files linked to a small number of articles. For example, 95% of all RefSNP citations appear in 5% of the articles.

**Table 4:** **Distribution of database citations in the supplementary data of the top 5% articles by databases**

Database	Total number of articles containing database citations in their supplementary data	% of database citations in the supplementary data of the top 5% articles
ENA	2,458	88.78%
PDBe	1,274	86.36%
RefSNP	1,167	95.05%
UniProt	1,059	83.87%
RefSeq	721	63.39%
Pfam	617	70.15%
InterPro	499	72.46%
Ensembl	377	67.62%
ArrayExpress	66	88.35%
OMIM	57	63.79%

## Conclusions

We extended our previous study on database citation in full text articles to supplementary data. We analysed and compared the distributions of database citations in article bodies and their supplementary data for ten different biological databases. Three of these databases are primary (ENA, ArrayExpress, PDBe) and the other seven databases (Pfam, Uniprot, RefSeq, RefSNP, Ensembl, InterPro, OMIM) are secondary databases. The main outcomes are as follows: (1) Only a small number of supplementary files contain accession numbers and in general these citations are skewed towards a small number of articles that contain large collections of accession numbers in their supplementary files. Not surprisingly, supplementary files contain much larger references of data points than article bodies. (2) In article bodies, the most frequently cited databases are ENA, PDBe and RefSNP whereas in supplementary files such databases are ENA, UniProt, RefSeq and RefSNP. This indicates that secondary databases are themselves also being reused as datasets for analysing and perhaps highlighting that there is no convenient way to cite these subsets of public databases. (3) It is possible that articles with accession numbers in supplementary data may be useful to curators, not only directly (i.e. as data points they may want to use in curation processes) but also as a means to highlight data-rich articles. Given that the shared accession numbers between article body and supplementary data is tiny, this richness is currently lost.

Our study highlights that supplementary data should be considered for text mining and indexing purposes in order to deliver search results that fully cover the content of the article. In addition, it shows that supplementary data is useful for the integration of literature and biomolecular databases as well as in curation tasks, where the details of cited data are important for accurate annotation of data records. There is a significant amount of heterogeneity in the provision of supplemental data - not only between journals but also between articles from the same title. Generally, the editor or author decide what to provide as supplementary data in what format. For example, the results from the analysis of microarray data are represented graphically in some supplementary files while they are represented in table format in some other supplementary files. Rendering the data represented in graphical form as leaves the information undiscoverable by any other means except reading the article, without the development of new tools.

There are a few emerging guidelines on what the supplemental data of articles should include (or not) and how, such as the recently launched PLoS Data Availability Policy (http://www.plosone.org/static/policies.action#sharing), but as yet these are not universally available. Efforts such as BioSharing (http://www.biosharing.org) are providing organizational focus to the challenge of managing supplemental data, but the impact of this and similar efforts is yet to be felt widely in the publishing community. What is clear is that there is a growing expectation regarding the management of supplemental data, including how and where to archive it and cite it in research articles. The recently formulated and widely endorsed Data Citation Principles (https://www.force11.org/datacitation) are an excellent step towards a more formal approach to data citation in research articles; although, how to cite large datasets that are subsets of public databases in a useful and elegant manner will require further work. Finally, this study, along with previous similar studies (6) demonstrates that all of the research outputs of an experiment are useful, and that all related information should be considered for inclusion in analyses or data extraction alongside the article narrative.

### Data citations

Şenay Kafkas, Jee-Hyub Kim, Xingjun Pi, Johanna R. McEntyre, 2014, Accession numbers in supplementary files, Zenodo, http://dx.doi.org/10.5281/zenodo.11771.

## Abbreviations

PMC: PubMed Central
OA: Open access
OA-ePMC: Open access subset of Europe PubMed Central
XML: eXtensible Markup Language
ENA: The European Nucleotide Archive
PDBe: Protein Data Bank, Europe
Pfam: The Protein families database
UniProt: Universal Protein knowledgebase
RefSeq: Reference sequence
RefSNP: Reference single nucleotide
OMIM: Online Mendelian Inheritance in Man InterPro.
